# Prediction of Vasoactive-Inotropic Score on Prolonged Mechanical Ventilation in Adult Congenital Heart Disease Patients After Surgical Treatment Combined with Coronary Artery Bypass Grafting

**DOI:** 10.21470/1678-9741-2023-0218

**Published:** 2024-05-13

**Authors:** Jia Liu, Yinghong Zhang, Wen Zhang, Juanzhou Hu, Pan Peng, Shiqi Zhou, Jing Huang, Jiangyun Peng

**Affiliations:** 1 Institute of Nursing Research, Hubei Province Key Laboratory of Occupational Hazard Identification and Control, School of Medicine, Wuhan University of Science and Technology, Wuhan, People’s Republic of China; 2 Department of Cardiac Surgery, Wuhan Asian Heart Hospital, Wuhan, People’s Republic of China; 3 Department of Cardiothoracic Surgery, Wuhan Puren Hospital, Wuhan, People’s Republic of China

**Keywords:** Adult, Congenital Heart Disease, Coronary Artery Bypass, Mechanical Ventilation, Vasoactive-inotropic score

## Abstract

**Introduction:**

This study aimed to investigate the predictive value of the vasoactive-inotropic score (VIS) at different time points for postoperative prolonged mechanical ventilation (PMV) in adult congenital heart disease patients undergoing surgical treatment combined with coronary artery bypass grafting.

**Methods:**

Patients were divided into two groups that developed PMV or not. The propensity score matching method was applied to reduce the effects of confounding factors between the two groups. VIS at different time points (VIS at the end of surgery, VIS_6h_, VIS_12h_, and VIS_12h max_) after surgery were recorded and calculated. The value of VIS in predicting PMV was analyzed by the receiver operating characteristic (ROC) curve, and multivariate logistic regression was used to analyze independent risk factors.

**Results:**

Among 250 patients, 52 were in the PMV group, and 198 were in the non-PMV group. PMV rate was 20.8%. After propensity score matching, 94 patients were matched in pairs. At each time point, the area under the ROC curve predicted by VIS for PMV was > 0.500, among which VIS at the end of surgery was the largest (0.805). The optimal cutoff point for VIS of 6.5 could predict PMV with 78.7% sensitivity and 72.3% specificity. VIS at the end of surgery was an independent risk factor for PMV (odds ratio=1.301, 95% confidence interval 1.091~1.551, P<0.01).

**Conclusion:**

VIS at the end of surgery is an independent predictor for PMV in patients with adult congenital heart disease surgical treatment combined with coronary artery bypass grafting.

## INTRODUCTION

**Table:** 

Abbreviations, Acronyms & Symbols				
ACC	= Aortic cross-clamping		NYHA	= New York Heart Association
ACHD	= Adult congenital heart disease		OR	= Odds ratio
ASA	= American Society of Anesthesiologists		PAH	= Pulmonary arterial hypertension
AUC	= Area under the curve		PCI	= Percutaneous coronary intervention
BMI	= Body mass index		PLT	= Platelet
CABG	= Coronary artery bypass grafting		PMV	= Prolonged mechanical ventilation
CI	= Confidence interval		PSM	= Propensity score matching
COPD	= Chronic obstructive pulmonary diseases		ROC	= Receiver operating characteristic
CPB	= Cardiopulmonary bypass		SE	= Standard error
ICU	= Intensive care unit		UA	= Uric acid
LOS	= Length of stay		VIS	= Vasoactive-inotropic score
LVEF	= Left ventricular ejection fraction		WBC	= White blood cell

According to the 2020 European Society of Cardiology Guidelines, > 90% of patients with congenital heart disease can survive to adulthood^[1]^. Moreover, 90% of mild, 75% of moderate, and 40% of complex adult congenital heart disease (ACHD) patients can live past 60 years^[1]^. However, ACHD patients are more likely to suffer from coronary artery disease^[2]^, which has been identified as a significant predictor of mortality for patients over 60 years^[3,4]^. Surgical treatment combined with coronary artery bypass grafting (CABG) has become one of the most effective therapies to cure these patients. Previous studies have focused on their immediate and long-term mortality^[3]^. Several studies have confirmed that vasoactive-inotropic score (VIS) can predict mortality and poor outcomes after surgery, such as cardiac arrest, mechanical circulatory support, renal replacement therapy, stroke, or seizure^[5-8]^. However, there are fewer studies on the predictive value of VIS for prolonged mechanical ventilation (PMV). Most patients who underwent open-heart surgery in China can be extubated within 24 hours after surgery^[9]^, while PMV was defined as ventilation ≥ 5 days in previous studies^[10,11]^. Therefore, this study focused on the investigation of the predictive value of VIS for PMV (> 48 hours) within 12 hours after surgery in Chinese ACHD patients who underwent surgical treatment and CABG. In addition, we tried to identify the optimal cutoff point of VIS for PMV in order to help medical staff find high-risk patients for PMV at the early stage.

## METHODS

The data of ACHD patients who underwent surgical treatment and CABG in Wuhan Asian Heart Hospital affiliated with Wuhan University of Science and Technology from March 2003 to October 2021 were retrospectively recorded via the hospital’s electronic medical record system. The study was approved by the Medical Ethics Committee of Wuhan University of Science and Technology (Ethics No.: 2022116).

Inclusion criteria were patients aged ≥ 18 years, patients with congenital heart disease diagnosed by echocardiography, patients diagnosed with coronary atherosclerotic heart disease, ACHD patients with surgical treatment combined with CABG, and patients admitted to intensive care unit (ICU) and who required mechanical ventilation. Exclusion criteria were patients who underwent cardiac malformation correction and CABG during different periods, patients with off-pump surgery, patients who died within 48 hours of surgery, and patients with incomplete data.

There were 39 variables recorded in this study: (1) general conditions - age, sex, body mass index (BMI), route of admission, history of chronic obstructive pulmonary diseases, pulmonary arterial hypertension, hypertension, diabetes mellitus, hyperlipidemia, cerebral ischemic stroke, atrial fibrillation, and history of percutaneous coronary intervention; (2) preoperative data - ACHD diagnosis, ACHD complexity classification, number of main coronary artery lesions, left ventricular ejection fraction, New York Heart Association classification, American Society of Anesthesiologists (ASA) classification, white blood cell count, neutrophil count, platelet count, uric acid, serum creatinine, albumin; (3) intraoperative data - cardiopulmonary bypass time, aortic cross-clamping time, and the maximum intraoperative lactate; (4) postoperative data - VIS at the end of surgery, VIS six hours after surgery, VIS 12 hours after surgery, maximum VIS within 12 hours after surgery, postoperative duration of mechanical ventilation, redo thoracotomy surgery, new-onset postoperative atrial fibrillation, postoperative pulmonary complications, neurological complications, acute kidney injury, ICU length of stay, and hospital length of stay. The laboratory data were collected within 24 hours of admission. Postoperative pulmonary complications occurred if a patient experienced at least one of the following: pneumonia, atelectasis detected, pleural effusion, or respiratory failure within seven days after surgery. Postoperative neurological complications were defined as temporary neurological dysfunction and permanent neurological dysfunction. Determination of pneumonia, atelectasis detected, pleural effusion, respiratory failure, neurological complications, and acute kidney injury were retrieved from the patient’s medical record.

VIS was calculated as the following formula^[12]^: VIS = dopamine dose (µg/kg/min) + dobutamine dose (µg/kg/min) + 10 × milrinone (µg/kg/min) + 100 × norepinephrine (µg/kg/ min) + 100 × epinephrine (µg/kg/min) + 10000 × vasopressin (U/kg/min). And VIS was calculated every hour. The vasoactive medication dose was readily adjusted according to the patient’s blood pressure and heart rate. Early postoperative use of vasoactive agents can avoid multi-organ ischemic dysfunction, and the dose needs to be discontinued or reduced promptly when the patient is circulatory stable. VIS was recorded as 0 if the abovementioned six medications were not used for the patients. All patients were divided into two groups based on the duration of postoperative mechanical ventilation, including the control group for PMV ≤ 48 hours and the PMV group for PMV > 48 hours^[13]^.

IBM Corp. Released 2019, IBM SPSS Statistics for Windows, version 26.0, Armonk, NY: IBM Corp. was used for statistical analysis. *P*-values are two-tailed, and *P*<0.05 was considered statistically significant. The continuous baseline data were expressed as the mean ± standard deviation and median (25^th^ percentile, 75^th^ percentile), and the categorial data were expressed as frequency (%). There was heterogeneity in ACHD patients with combined coronary artery disease, and propensity score matching can minimize the bias of baseline characteristics and balance confounding effects between the two groups. So, we matched patients with the following factors as covariates: age, sex, BMI, route of admission, pulmonary arterial hypertension, ACHD complexity classification, number of main coronary artery lesions, and ASA classification. Propensity score matching was performed on a 1:1 basis using the nearest neighbor matching method with a caliper value of 0.01. The *t*-test, chi-square test, Fisher’s exact test, and Mann-Whitney U test were used for the univariate analysis. Multivariate logistic regression analysis was performed for parameters with statistical significance in univariate analysis. The predictive value of VIS was evaluated using receiver operating characteristic (ROC) curve, and cutoff values were generated based on the maximum Youden index to calculate sensitivity and specificity.

## RESULTS

### Patients’ Characteristics

Among 266 ACHD patients who underwent surgical treatment combined with CABG, seven cases of non-simultaneous surgery, two cases of off-pump surgery, one case of death within 48 hours after surgery, and six cases of incomplete data were excluded, then 250 patients were finally included. There were 162 (64.8%) males and 88 (35.2%) females, aged 36-81 years, with an average age of 58 years.

The most common diagnosis was atrial septal defect (n=133, 53.2%), followed by the bicuspid aortic valve (n=43, 17.2%), ventricular septal defect (n=30, 12%), quadricuspid aortic valve (n=1, 0.4%), coronary artery fistula (n=11, 4.4%), anomalous origin of coronary artery (n=5, 2%), partial endocardial cushion defect (n=4, 1.6%), double-chambered right ventricle (n=5, 2%), Ebstein anomaly (n=4, 1.6%), pulmonary valve stenosis (n=3, 1.2%), patent ductus arteriosus (n=2, 0.8%), partial anomalous pulmonary venous connection (n=2, 0.8%), unroofed coronary sinus syndrome (n=2, 0.8%), congenital descending aortic stenosis (n=1, 0.4%), congenital aortic-left ventricular tunnel (n=1, 0.4%), tetralogy of Fallot (n=1, 0.4%), sinus of Valsalva aneurysm (n=1, 0.4%), and left triatrial heart (n=1, 0.4%).

All surgical interventions for congenital heart diseases included: atrial septal defects and ventricular septal defects treated with direct suture closure (n=90, 36%) and patch repair (n=73, 29.2%), bicuspid aortic valve and quadricuspid aortic valve treated with aortic valvuloplasty (n=2, 0.8%) and aortic valve replacement (n=42, 16.8%), partial endocardial cushion defect treated with mitral valvuloplasty and defect repair (n=4, 1.6%), pulmonary valvuloplasty for pulmonary valve stenosis (n=3, 1.2%), patent ductus arteriosus (n=2, 0.8%) and congenital coronary artery fistula treated with surgical closure (n=11, 4.4%), and other congenital heart diseases treated with corresponding corrective surgeries (n=23, 9.2%).

The vasoactive medications used in this study were dopamine (n=199, 79.6%), dobutamine (n=156, 62.4%), norepinephrine (n=58, 23.2%), epinephrine (n=32, 12.8%), and milrinone (n=2, 0.8%).

### Propensity Score Matching

After matching, the covariates such as age, sex, BMI, route of admission, pulmonary arterial hypertension, ACHD complexity classification, number of main coronary artery lesions, and ASA classification between the PMV group and the non-PMV group were balanced, as shown in Table 1. A total of 94 patients were included in the study, and then the data was analyzed.

**Table 1:** Balance comparisons of covariates between the PMV group and the non-PMV group after PSM.

Variable	Before matching			After matching		
	PMV group (n=52)	Non-PMV group (n=198)	*P*-value	PMV group (n=47)	Non-PMV group (n=47)	*P*-value
Age (year)	59.27 ± 7.88	57.51 ± 8.79	0.191	59.13 ± 8.13	58.13 ± 7.74	0.543
Sex (male)	36 (69.2)	126 (63.6)	0.452	31 (66.0)	32 (68.1)	0.826
BMI (kg/m^2^)	23.32 ± 2.97	24.44 ± 3.30	0.027	23.51 ± 2.90	23.08 ± 2.88	0.473
Emergency admission (%)	11 (21.2)	40 (20.2)	0.880	8 (17.0)	7 (14.9)	0.778
PAH (%)	21 (40.4)	57 (28.8)	0.108	18 (38.3)	20 (42.6)	0.674
ACHD Complexity Classification (%)			0.960			0.466
Mild	36 (69.2)	133 (67.2)		32 (68.1)	29 (61.7)	
Moderate	12 (23.1)	49 (24.7)		11 (23.4)	10 (21.3)	
Severe	4 (7.7)	16 (8.1)		4 (8.5)	8 (17.0)	
Number of main coronary artery lesions (%)			0.610			0.631
Single-branch lesion	15 (28.8)	70 (35.4)		14 (29.8)	18 (38.3)	
Double-branch lesions	13 (25.0)	40 (20.2)		12 (25.5)	12 (25.5)	
Triple-branch lesions	24 (46.2)	88 (44.4)		21 (44.7)	17 (36.2)	
ASA Classification (%)			0.032			0.489
2	5 (9.6)	21 (10.6)		5 (10.6)	4 (8.5)	
3	37 (71.2)	166 (83.8)		35 (74.5)	40 (85.1)	
4	10 (19.2)	11 (5.6)		7 (14.9)	3 (6.4)	

The results of continuous variables are expressed as mean ± standard deviation for those with normal distribution, and categorical data were expressed as numbers (%)

ACHD=adult congenital heart disease; ASA=American Society of Anesthesiologists; BMI=body mass index; PAH=pulmonary arterial hypertension; PMV=prolonged mechanical ventilation; PSM=propensity score matching

### Predictive Value of VIS for PMV

**Fig. 1:**
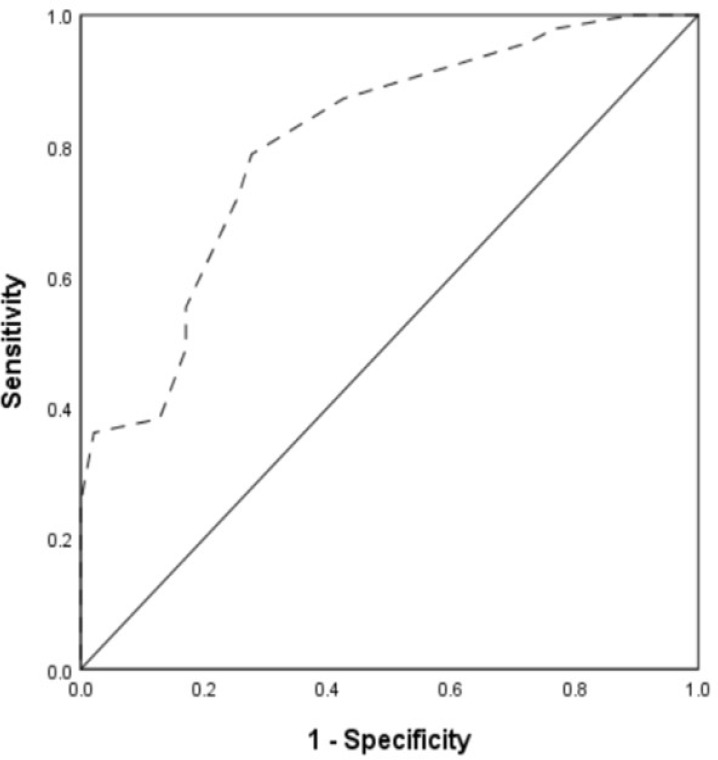
Receiver operating characteristics (ROC) curve for vasoactive inotropic score at the end of surgery - area under the ROC (95% confidence interval): 0.805(0.718-0.892) (P<0.01).

To predict PMV, the area under the ROC curve of VIS at the end of surgery, six hours after surgery, 12 hours after surgery, and maximum VIS within 12 hours after surgery was > 0.500 (*P*<0.05), with VIS at the end of surgery having the largest area under the curve (AUC=0.805) (Table 2). According to the Youden index formula, the optimal cutoff point of VIS at the end of surgery was 6.5, with a sensitivity of 78.7% and a specificity of 72.3% (Figure 1).

**Table 2:** Receiver operating characteristic curve for VIS.

Variable	AUC	SE	*P*-value	95% CI
VIS at the end of surgery	0.805	0.044	< 0.01	0.718-0.892
VIS_6h_	0.707	0.053	< 0.01	0.603-0.810
VIS_12h_	0.706	0.053	< 0.01	0.603-0.810
VIS_12h max_	0.733	0.051	< 0.01	0.633-0.833

AUC=area under the curve; CI=confidence interval; SE=standard error; VIS=vasoactive-inotropic score

### Independent Risk Factors for PMV

Univariate analysis showed significant differences in atrial fibrillation, preoperative left ventricular ejection fraction, serum uric acid, cardiopulmonary bypass time, aortic cross-clamping time, the highest value of intraoperative lactate, and VIS at the end of surgery between the two groups (*P*<0.05) (Table 3). The multivariate logistic regression analysis model included VIS at the end of surgery and other variables with univariate analysis (*P*<0.05) (Model 1). After controlling for uric acid and cardiopulmonary bypass time, VIS at the end of surgery (odds ratio [OR]=1.301, 95% confidence interval [CI] 1.091~1.551, *P*<0.01) was an independent risk factor for PMV after surgical treatment combined with CABG (Table 4). Patients were divided into high and low VIS group according to the optimal cutoff point of 6.5 for VIS at the end of surgery. The same factors in Model 1 were again included in the logistic regression analysis model (Model 2), and the results showed that VIS at the end of surgery (OR=9.067, 95% CI 2.961~27.762, *P*<0.001) was an independent risk factor for PMV among patients after controlling for uric acid and cardiopulmonary bypass time (Table 4).

**Table 3:** Clinical characteristics of research groups.

Variable	PMV group (n=47)	Non-PMV group (n=47)	*P*-value
COPD (%)	0 (0)	2 (4.3)	0.495
Hypertension (%)	22 (46.8)	21 (42.6)	0.678
Diabetes (%)	8 (17.0)	11 (23.4)	0.441
Hyperlipidemia (%)	7 (14.9)	8 (17.0)	0.778
Cerebral ischemic stroke (%)	13 (27.7)	6 (12.8)	0.072
Atrial fibrillation (%)	17 (36.2)	7 (14.9)	0.018
History of PCI (%)	0 (0)	2 (4.3)	0.495
NYHA ≥ 3 (%)	14 (29.8)	10 (21.3)	0.344
LVEF (%)	54 (50, 56)	56 (52, 60)	0.024
WBC count (^×^ 10^9^/L)	6.08 (4.60, 7.70)	5.55 (4.66, 7.06)	0.281
PLT count (^×^ 10^9^/L)	178.10 (119.20, 255.00)	164.00 (137.00, 204.50)	0.748
UA (µmol/L)	407 (322, 513)	338 (280, 394)	0.001
Serum creatinine (mmol/L)	74.12 (67.00, 92.00)	74.12 (62.00, 82.00)	0.427
Albumin (g/L)	39.74 ± 3.73	40.71 ± 3.54	0.198
CPB time (min)	157 (122, 227)	117 (97, 140)	< 0.001
ACC time (min)	97 (72, 139)	72 (55, 85)	< 0.001
Lactate (mmol/L)	2.20 (1.40, 3.00)	1.70 (1.10, 2.35)	0.034
VIS at the end of surgery	9 (7,15)	5 (4, 8)	< 0.001
VIS_6h_	8 (5, 18)	5 (3, 8)	0.001
VIS_12h_	9 (5, 25)	5 (3, 10)	0.001
VIS_12h max_	8 (11, 35)	7 (5, 11)	< 0.001

The results of continuous variables are expressed as mean ± standard deviation for those with normal distribution, median (25^th^ percentile and 75^th^ percentile) for those with skewed distribution, and categorical data were expressed as numbers (%). Comparisons between the two groups were performed by Student *t*-tests for continuous variables with normal distribution and by non-parametric Mann-Whitney U test for continuous variables with skewed distribution. Categorical data analysis was performed by Chi-square tests or Fisher’s exact tests

ACC=aortic cross-clamping; COPD=chronic obstructive pulmonary diseases; CPB=cardiopulmonary bypass; LVEF=left ventricular ejection fraction; NYHA=New York Heart Association; PCI=percutaneous coronary intervention; PLT=platelet; PMV=prolonged mechanical ventilation; UA=uric acid; VIS=vasoactive-inotropic score; WBC=white blood cell

**Table 4:** Association between VIS at the end of surgery and PMV.

Variable	Multivariate regression					
	Model 1			Model 2		
	OR	95% CI	*P*-value	OR	95% CI	*P*-value
UA (µmol/L)	1.006	1.001~1.012	0.028	1.006	1.001~1.012	0.018
CPB time (min)	1.014	1.002~1.026	0.017	1.018	1.006-1.030	0.004
VIS at the end of surgery	1.301	1.091~1.551	0.003	9.067	2.961~27.762	< 0.001

A forward stepwise multivariate logistic regression was used to evaluate the associated factors

CI=confidence interval; CPB=cardiopulmonary bypass; OR=odds ratio; PMV=prolonged mechanical ventilation; UA=uric acid; VIS=vasoactive-inotropic score

### Patients’ Clinical Outcomes

Univariate analysis showed that differences in postoperative pulmonary complications, neurological complications, acute kidney injury, ICU length of stay, and hospital length of stay between the two groups were significant (*P*<0.05) (Table 5).

**Table 5:** Clinical outcomes of research groups.

Variable	PMV group (n=47)	Non-PMV group (n=47)	*P*-value
Redo thoracotomy surgery	6 (12.8)	2 (4.3)	0.267
New-onset postoperative atrial fibrillation	10 (21.3)	7 (14.9)	0.421
Postoperative pulmonary complications	47 (100.0)	20 (42.6)	< 0.001
Neurological complications	12 (25.5)	3 (6.4)	0.011
Acute kidney injury	20 (42.6)	8 (17.0)	0.007
ICU LOS (h)	135.17 (103.66, 186.83)	64.33 (48.00, 69.00)	< 0.001
Hospital LOS (d)	26 (23, 33)	21 (18, 25)	< 0.001

The results of continuous variables are expressed as median (25^th^ percentile and 75^th^ percentile) for those with skewed distribution, and categorical data were expressed as numbers (%). Comparisons between the two groups were performed by non-parametric Mann-Whitney U test for continuous variables with skewed distribution. Categorical data analysis was performed by Chi-square tests

ICU=intensive care unit; LOS=length of stay; PMV=prolonged mechanical ventilation

## DISCUSSION

Despite the tremendous progress in the perioperative management of cardiac surgery, the incidence of PMV in postoperative patients is still as high as 22%^[13]^, resulting in lung injury, other complications, and prolonged ICU or hospital stay. Additionally, some researchers claimed that the mortality rate of PMV patients reaches 40%^[14]^, which significantly increases the economic burden on patients. Therefore, PMV after cardiac surgery is currently an urgent issue to be focused.

In this study, we matched patients between the PMV group and the non-PMV group to standardize the patients with adult congenital diseases. Then, we found that VIS had a good predictive value for PMV (AUC > 0.500) at different time points, with VIS at the end of surgery having the largest AUC (0.805). The cutoff point of VIS at the end of surgery was 6.5, with a sensitivity of 78.7% and specificity of 72.3%. Multivariate logistic regression analysis showed that VIS at the end of surgery was an independent risk factor for PMV in patients undergoing ACHD surgery combined with CABG. Furthermore, the risk of PMV was significantly increased in patients with VIS at the end of surgery ≥ 6.5, up to 9-fold. This study also showed that postoperative PMV might be related to pulmonary complications, neurological complications, acute kidney injury, and ICU and hospital stay.

Although the patients maintain stable hemodynamics status at the end of surgery, their cardiac function has not yet fully recovered, so inotropic agents are necessary to improve tissue perfusion and prevent cardiopulmonary insufficiency and multi-organ failure^[14]^. After cardiac surgery, vasoactive medications are associated with impaired lung structure and function, such as increased vascular permeability and pulmonary edema. However, patients with reduced cardiac function after surgery require vasoactive medications to improve cardiac function and mechanical ventilation to improve total oxygen supply^[15]^. The study among pediatric patients with septic shock found that VIS was an independent risk factor for the length of ventilation and ICU stay^[15]^. Another study in children with congenital heart disease who underwent extracorporeal circulation found that VIS ≥ 10 between 24 and 48 hours after extracorporeal circulation was associated with longer postoperative mechanical ventilation, ICU stay, and days in hospital^[16]^. Hence, VIS may be related to the duration of mechanical ventilation in patients after cardiac surgery.

Because of differences in study populations and designs, the time points and the cutoff values for VIS to predict outcomes may be variable. The cutoff of VIS_48h_ in our study was lower than that of the previous study (6.5 *vs.* 10.5)^[17]^. That may be because 70 infants had respiratory hypoplasia, and 39.1% of infants had delayed chest closure, so the postoperative mechanical ventilation time was more extended than that in adults^[17]^. Yamazaki et al.^[18]^ demonstrated that VIS at the end of surgery predicted adverse outcomes in 129 adult patients undergoing cardiopulmonary bypass surgery (AUC=0.77), and patients with VIS at the end of surgery > 5.5 experienced longer ICU stay and longer ventilation. In addition, Baysal et al.^[19]^ pointed out that VIS at the end of surgery > 5.5 could also predict mortality and morbidity (AUC=0.969), such as mechanical circulatory support, cardiac arrest, and arrhythmia in CABG patients. In this study, the cutoff value of VIS at the end of surgery was higher than those in the abovementioned two researches, which may be because the cardiovascular function of ACHD patients who underwent surgical treatment combined with CABG is worse, causing more requirement for vasoactive medications to maintain hemodynamic stability.

As a predictor, VIS can be updated in time for early postoperative mortality and morbidity risk prediction by simple calculation^[20]^. Although lactate has been associated with adverse outcomes in cardiovascular surgery, Kim et al.^[21]^ noted that there was no significant relation between postoperative lactate levels and duration of mechanical ventilation in ACHD patients; similar results were obtained in our study. Additionally, the cardiac index and mixed venous oxygen saturation output obtainments rely on the pulse index continuous cardiac output system, but VIS does not. Thus, VIS may be more widely available and less dependent on healthcare professionals and instrumentations. As a result, VIS may be more rapid and straightforward in predicting postoperative outcomes when compared to other evaluation indicators.

### Limitations

There were some limitations to this study. Firstly, because the sample was recorded from a single center, it is essential to further conduct multicenter studies in the future. Secondly, due to the differences between each sort of ACHD operation, the predictive value of VIS and its cutoff value may vary. Thirdly, there may be some procedure variations due to differences in surgical teams.

## CONCLUSION

VIS at the end of surgery is an independent predictor for PMV in patients with ACHD surgical treatment combined with CABG. Therefore, healthcare specialists can use VIS to predict the risk of PMV, guide clinical decision-making, and improve patients’ prognoses.
